# Comparison of gene expression profiles in core biopsies and corresponding surgical breast cancer samples

**DOI:** 10.1186/bcr1542

**Published:** 2006-08-18

**Authors:** Rosanna Zanetti-Dällenbach, Vincent Vuaroqueaux, Edward Wight, Martin Labuhn, Gad Singer, Patrick Urban, Urs Eppenberger, Wolfgang Holzgreve, Serenella Eppenberger-Castori

**Affiliations:** 1 Women's University Hospital Basel, Switzerland; 2 Stiftung Tumorbank Basel, Switzerland; 3 OncoScore AG, Riehen, Switzerland; 4 Department of Pathology, University Hospital Basel, Switzerland

## Abstract

**Introduction:**

Gene expression profiling has been successfully used to classify breast cancer into clinically distinct subtypes, and to predict the risk of recurrence and treatment response. The aim of this study was to investigate whether the gene expression profile (GEP) detected in a core biopsy (CB) is representative for the entire tumor, since CB is an important tool in breast cancer diagnosis. Moreover, we investigated whether performing CBs prior to the surgical excision could influence the GEP of the respective tumor.

**Methods:**

We quantified the RNA expression of 60 relevant genes by quantitative real-time PCR in paired CBs and surgical specimens from 22 untreated primary breast cancer patients. Subsequently, expression data were compared with independent GEPs obtained from tumors of 317 patients without preceding CB.

**Results:**

In 82% of the cases the GEP detected in the CB correlated very well with the corresponding profile in the surgical sample (r_s _≥ 0.95, p < 0.001). Gene-by-gene analysis revealed four genes significantly elevated in the surgical sample compared to the CB; these comprised genes mainly involved in inflammation and the wound repair process as well as in tumor invasion and metastasis.

**Conclusion:**

A GEP detected in a CB are representative for the entire tumor and is, therefore, of clinical relevance. The observed alterations of individual genes after performance of CB deserve attention since they might impact the clinical interpretation with respect to prognosis and therapy prediction of the GEP as detected in the surgical specimen following CB performance.

## Introduction

Gene expression profiling by parallel detection of thousands of genes permits the molecular signature (phenotype) of a tissue sample to be read and can, therefore, individually characterize a patient's tumor at the molecular level. Based on the gene expression profile (GEP) of a tumor, a molecular classification for breast cancer was proposed [1] and several molecular signatures were reported to predict the risk of recurrence and treatment response [2-5]. Such molecular analyses require only small amounts of material, such as tissue samples obtained by minimal invasive methods, for example, core biopsy (CB), which are used to assess the nature of palpable and non-palpable breast lesions to confirm or exclude the diagnosis of breast cancer [6-9].

Although CB investigations have become more and more important in the early workup of breast lesions, there are only a few investigations regarding the reliability of GEPs as detected in CBs [10]. However, wound healing subsequent to CB can potentially induce gene expression alterations in the injured tissue. Many of these normally occurring reparative processes share molecular characteristics with an aggressive cancer phenotype, such as cell proliferation and survival, angiogenesis, and extracellular matrix remodeling; these biological hallmarks were shown to predict the clinical course in cancer [3,11-13]. Therefore, potential biological changes induced by CB require further study since they may have important consequences for clinical outcome prediction and treatment decisions as well as the interpretation of GEP changes investigated in neo-adjuvant studies.

We explored the molecular expression levels of 60 genes using quantitative real-time PCR (qrt-PCR), a highly sensitive and reproducible method, in paired CB and surgical samples. These genes were selected according to their known links to malignant cell behavior in breast cancer and their importance in major cancer hallmarks, such as proliferation, survival, invasiveness and angiogenic potential, and in the wound healing process [14]. Our first objective was to investigate whether the molecular profile of a CB is representative for the whole tumor. The second objective was to study if the repair process following CB alters the GEP and if this is influenced by the timeframe between the CB and the surgical excision.

## Materials and methods

### Tumor specimen acquisition

Between June 2004 and June 2005, 22 consecutive breast cancer patients entered this study, for which both CBs and surgical tumor tissue specimens (STs) were available. This study was undertaken at the Women's University Hospital Basel, Switzerland, and approved by the local institutional review board (EKBB permission Nr. 81/04). Written informed consent was obtained from all patients.

All CBs (14-gauge needle, Magnum^® ^Core high speed, Bard Medica, Karlsruhe, Germany) were obtained under sonographic guidance (HDI 5'000 Sono CT^®^, Philips, Zurich, Switzerland) under local anesthesia through a skin incision in a sterile field. Five biopsy specimens were taken routinely for each patient. Two biopsy specimens were divided longitudinally; two halves to be used for molecular examination were, within one minute, stored in RNAlater^®^-solution (Qiagen, Basel, Switzerland), while the other two halves as well as the other three biopsies were immediately put in formaldehyde and sent for histological examination to the Institute of Pathology, University Hospital Basel.

After establishing the diagnosis of breast cancer, all patients underwent breast surgery with sentinel node lymphonodectomy or axillary lymphonodectomy if indicated. All surgical samples were examined by the same pathologist. If the tumor tissue was larger than 0.5 cm in diameter at the intra-operative frozen section, a representative piece containing more than 60% tumor cells was cryopreserved within five minutes and made available to the Stiftung Tumorbank Basel for molecular examination. The rest of the tumor tissue was embedded in paraffin for routine histological examination.

### Reference study population

RNA expression levels of all 60 genes were detected using the same qrt-PCR method in 317 surgically excised breast cancer specimens [15] from patients undergoing primary surgery in 1992 to 1996 without previous examination by CB. All tissue samples were prepared by the pathologists as described above, all samples contained more than 60% tumor cells and they were cryoperserved within five minutes. The Stiftung Tumorbank Basel was subsequently responsible for collection, storage at -70°C and analysis.

### RNA extraction and qrt-PCR

Detailed procedures have been published elsewhere [16]. In brief, RNA was extracted using the RNeasy Mini Kit (Qiagen, Inc., Valencia, CA, USA), quantified and quality-checked on a Bioanalyzer 2100 (RNA 6000 Nano LabChip-Kit, Agilent Technologies, New Castle, DE, USA). High quality RNA samples were reverse-transcribed (10 mM DDT, 1 μg of hexamer primers, 2 U of MMLV Reverse Transcriptase (Invitrogen, Basel, Switzerland), 40 U of RNasin (Promega, Wallisellen, Switzerland), 0.5 mM of each dNTP (Promega), 1× reaction buffer). PCR primers were designed to be cDNA specific and ordered at GeneScan Europe (Freiburg, Germany). PCR was performed in 40 cycles on an ABI Prism 7000 using 2× SYBR Green I Master Mix (Applied Biosystems, Forster City, CA, USA) in a final volume of 25 μl. Relative quantities (ΔCt) were obtained by normalization against ribosomal 18S RNA, and standardization was achieved with Human Universal Standard RNA (Stratagene Europe, Amsterdam, The Netherlands). The 60 genes quantitatively assessed are listed in Table 1.

### Statistical analysis

The same amount of RNA was used for the GEP analysis of each sample. For statistical analysis, ΔCt expression values of each gene were obtained by normalizing the raw gene values to 18S rRNA as a reference gene.

Cluster and TreeView programs were used to perform unsupervised hierarchical clustering of samples and genes (Spearman correlation, average linkage) [17]. Spearman correlation coefficients were calculated to compare the GEPs of all paired samples. Differentially expressed genes were identified with the paired two-sample *t*-test. The Mann-Whitney *U*-test was used to compare median expression values of genes among different subgroups of patients. All statistical analyses were carried out at 5% level of significance and performed with S-Plus software (Version 6.1, Insightful Corporation, Seattle, WA, USA).

## Results

### Patient characteristics

The mean age of the 22 patients was 63 years (range: 34 to 81 years). The period between CB and final surgery ranged from 1 to 23 days. The mean tumor diameter was 2.9 cm (range: 0.9 to 9 cm). Clinicopathological characteristics of each patient are listed in Table 1. Of note, one paired tissue sample was not further evaluated due to poor RNA quality, and RNA extracted from the two halves of the CB was not pooled in three cases but analyzed separately.

### Comparison of the gene expression profile in paired samples

**Figure 1:**
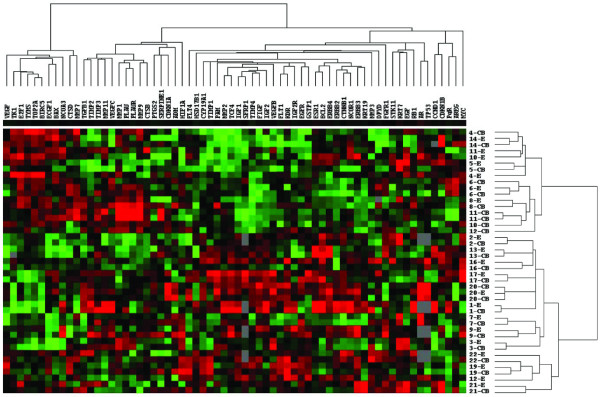
Unsupervised hierarchical clustering of paired core biopsy and surgical tumor tissue samples (Spearman correlation, average linkage). Red color indicates high expression levels (low ΔCt) and green vice versa. Patients 6, 11 and 20 had two core biopsies taken that were analyzed separately. In four cases the paired gene expression profiles did not cluster together close to each other.

As shown in Figure 1, unsupervised hierarchical clustering revealed that paired CB and ST generally clustered together; in only four cases (patients 4, 10, 11, and 12) did the GEP of the CB not agglomerate with the profile of the respective ST. Interestingly, the two separate CBs taken from patient 11 were very similar to each other, although they differed from their ST GEP. The gene dendrogram of the cluster analysis also revealed that samples agglomerated in two main groups according to their respective estrogen receptor status, reconfirming the representative value of this study population.

**Figure 2:**
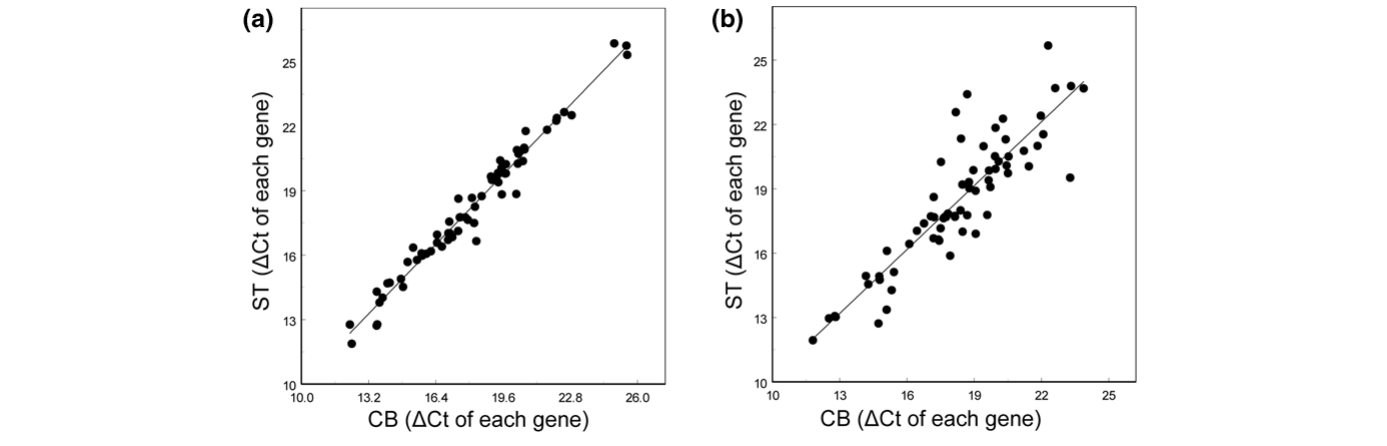
Correlation between the gene expression profiles (60 genes) of paired core biopsies (CB) and surgical tumor tissue specimens (ST). **(a) **Specimens from patient 2 (rs = 0.98), as representative for 82% of all cases. **(b) **Specimens from patient 12, as representative for less correlated paired gene expression profiles (rs = 0.86).

Subsequent analysis of paired CBs and STs confirmed the high correlation between all samples (r_s _from 0.86 to 0.98, all p < 0.001) for each patient. A scatter plot of two representative examples of a paired CB/ST is displayed in Figure 2. The differences in paired GEPs does not seem to be related to the timeframe between CB and surgery or to any other clinicopathological parameters (Table 1).

### Gene-by-gene analysis in core biopsies and paired surgical specimens

**Figure 3:**
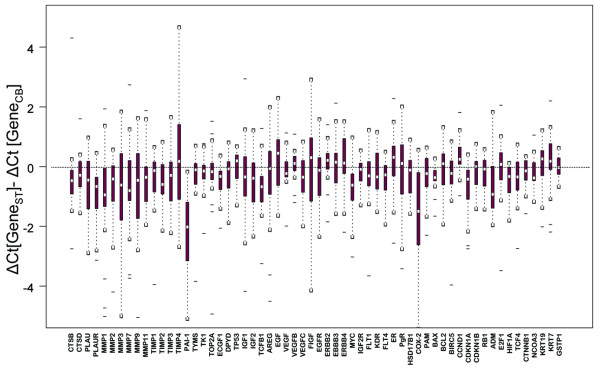
Box plots displaying the changes in the expression of each gene in the surgical specimens (ST) compared to the respective core biopsies (CB).

The comparison of the expression levels of individual genes by means of paired *t*-test showed no significant difference between CB and ST with the exception of four genes. Plasminogen activator inhibitor 1 (PAI-1; also known as SERPINE1) was significantly higher expressed (lower ΔCt values) in STs compared to CBs (p < 0.001, Table 1). Similar differences, although less pronounced, were observed for cyclooxygenase 2 (COX-2; also known as PTGS2; p < 0.001), urokinase plasminogen activator receptor (uPAR; also known as PLAUR; p = 0.003) and matrix metalloproteinase 1 (MMP1; p = 0.03). The increase in the expression of these genes was not related to the timeframe between CB and surgery. All other genes were very similarly expressed in paired CB/ST as shown in Figure 3. Table 1 lists differences in RNA expression values of PAI-1, COX-2 and ERBB2 for paired samples of each patient.

Histological re-examination of cryocuts of the surgical specimens revealed a certain amount of inflammation and fibrolysis. Whether these observations are due to cancerogenesis or to a *de novo *induced wound repair process can not be determined.

### Comparison of the expression levels of selected genes with a reference study population of surgical tumor tissue specimens

**Figure 4:**
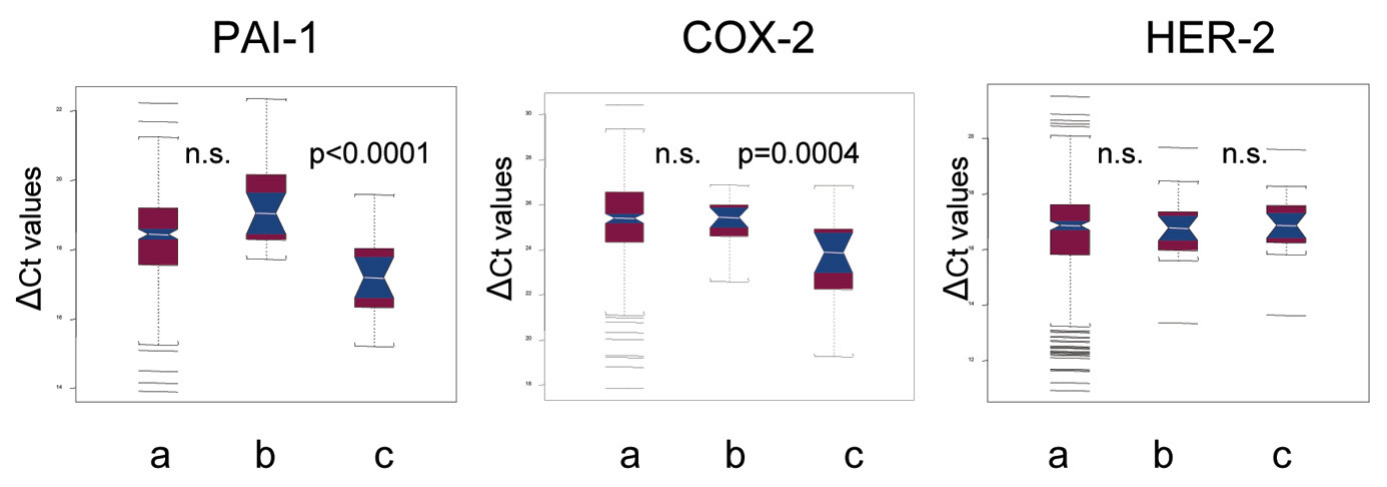
Notch box plots of the expression levels of plasminogen activator inhibitor (PAI)-1, cyclooxygenase (COX)-2 and HER-2 as detected **(a) **in 317 independent surgical specimens of primary breast cancer patients who did not undergo previous diagnosis by core biopsy, **(b) **in the core biopsy of the 21 patients entering this study, and **(c) **in the corresponding surgical excisions.

To verify whether the higher expression levels of PAI-1 and COX-2 observed in STs could have been induced by the preceding CB procedure, we compared the expression levels of the same genes in an independent population of 317 primary breast cancer patients. These samples were investigated with the same qrt-PCR technique but were from patients from which no CB had been taken prior to surgery. As shown in Figure 4, the expression levels of PAI-1 and COX-2 measured in the independent STs without CB were found to be very similar to the levels detected in CBs and significantly different from those detected in STs after CB. Moreover, no variation at all was observed for the remaining genes as illustrated by the expression levels of ERBB2 as an example.

## Discussion

Ultrasound-guided CB is a well established method to diagnose breast cancer in women, since it is a reliable, and time- and cost-saving method. The clinical utility of the information gained by CB depends on whether the CB is representative for the whole tumor. Our data demonstrate that the quantitative expression levels of 60 genes detected in CBs were highly comparable to their paired STs in 17 out of the 21 cases investigated. Even in the cases where GEPs of a CB and ST did not agglomerate, the expression levels of the ER and progesterone receptor as well as ERBB2 measured in the CB were also representative for the whole tumor. This is important since today's therapy decisions are based on these markers obtained either by CB or ST (St Gallen consensus recommendations [18]). In addition, our results reconfirm previous observations reported by immunohistochemistry [19,20] or semi-qrt-PCR [21].

Tissue sampling by CB causes a local injury, inducing wound healing that is characterized by recruitment of inflammatory cells, stimulation of stromal and epithelial cell proliferation, cell migration and increased angiogenesis. Analysis on a gene-by-gene basis demonstrated higher expression levels of PAI-1, COX-2, uPAR and MMP1 in STs compared to their paired CBs, whereas no changes were observed for all other genes. These results are not surprising since proteinases (such as PAI-1 and uPAR) are known to be essentially involved in the wound healing process [22,23] and COX-2 plays roles in inflammation and angiogenesis [24-27].

However, many of these reparation processes show parallels with cancerogenesis [28-30]; while proteinases, their inhibitors, cyto-/chemokines and growth factors are essential for wound healing and tissue repair, they also play central roles in cancer progression. For example, uPA, uPAR and its inhibitor PAI-1 are responsible for the degradation and remodeling of the extracellular matrix, and are further involved in angiogenesis, cell adhesion and migration necessary for tumor cell invasion and metastasis [31,32]. COX-2 can be induced by cytokines and growth factors during the inflammatory repair process as well as in cancer [24-26,30] resulting in COX-2 overexpression observed in human malignancies [25-27].

Therefore, increased levels of these markers in the tumor specimen could suggest a more aggressive cancer phenotype. Indeed, elevated levels of uPA and PAI-1 are associated with poor clinical outcome in breast cancer and also have predictive value [33-35]. Moreover, a previously identified 'wound-response signature' turned out to be prognostic in several carcinomas, including breast cancer [12,13]. Although COX-2 has been associated with increased Vascular Endothelial Growth Factor A (VEGF), estrogen synthesis, proliferation, apoptosis and invasion [25,27,36], in our study, higher levels of COX-2 were not accompanied by changes in the expression of genes involved in these processes, indicating that the observed molecular alterations influence data interpretation but not tumor aggressiveness.

## Conclusion

Our study demonstrates that expression levels of ER, progesterone receptor, ERBB2 and other genes relevant for the management of breast cancer as detected in CBs are representative for the whole tumor. However, increased expression levels of proteinases (e.g. PAI-1, uPAR, MMP1) and COX-2 in STs compared to their paired CBs suggest induction of theses genes during the repair process following tissue injury caused by CBs. This observation is important since such molecular alterations may have an impact on the clinical interpretation of GEPs detected in STs with respect to the prediction of risk assessment and treatment response.

## Abbreviations

CB = core biopsy; COX = cyclooxygenase; Ct = cycle threshold; GEP = gene expression profile; MMP = matrix metalloproteinase; qrt-PCR = quantitative real-time PCR; PAI = plasminogen activator inhibitor; PAI-1 = Plasminogen Activator Inhibitor-1; ST = surgical tumor tissue specimen; uPAR = urokinase plasminogen activator receptor; VEGF = Vascular Endothelial Growth Factor A.

## Competing interests

The authors declare that they have no competing interests.

## Authors' contributions

RS and SE designed the study, presented it to the ethical committee and drafted the manuscript. RS performed the sonographic guided core biopsies and coordinated the clinical part of the study. VV and ML contributed to the selection of the genes, selected primers and supervised RNA extraction and qrt-PCR. VV and PU contributed to statistical analysis, data interpretation and manuscript revision. GS performed the pathological analysis of all samples, prepared the surgical samples for molecular analysis and participated in data interpretation. EW, UE and WH participated in designing the study and writing the manuscript. SE coordinated the molecular investigation and performed statistical analysis. All authors read and approved the final manuscript.

## Figures and Tables

**Table 1:** Clinicopathological characteristics and gene expression variation of each patient

								**Difference in gene expression between CB and ST (ΔCt [Gene_ST_] – ΔCt [Gene_CB_])**		
**Patient number**	**Age**	**Time between CB and ST (days)**	**Histology**	**Tumor diameter (cm)**	**Grade**	**Lymph node involvement**	**Correlation (r_s_) between paired CB and ST**	**PAI-1**	**COX-2**	**ERBB2**

1	68	7	Ductal	3.3	3	Negative	0.97	-0.39	-0.15	-0.08
2	59	8	Ductal	1.8	3	Negative	0.98	-1.68	-0.20	0.52
3	49	21	Ductal	3.3	2	Positive	0.98	-1.19	-1.03	0.62
4	57	13	Ductal	1.1	1	Positive	0.89	-0.63	0.61	-0.02
5	63	14	Ductal	9.0	2	Negative	0.97	-1.41	-1.80	1.40
6	60	21	Ductulo-lobular	2.7	1	Positive	0.98	-1.38	-2.41	0.23
7	80	12	Ductal	3.1	1	Positive	0.95	-2.62	-1.39	0.44
8	81	14	Lobular	2.8	2	Positive	0.94	-3.78	-3.73	0.29
9	34	14	Ductal	1.8	2	Negative	0.95	-3.16	-1.37	0.12
10	68	10	Ductal	0.9	2	Micrometastasis	0.87	-3.57	-7.27	1.01
11	65	16	Lobular	1.9	1	Negative	0.89	-5.16	-0.80	-0.02
12	80	13	Ductal	6.0	3	Positive	0.86	-2.16	-0.20	0.63
13	59	2	Apocrine	2.7	3	Negative	0.98	-1.18	-4.64	-0.27
14	73	12	Lobular	1.9	2	Positive	0.95	-4.60	-3.97	-1.85
15	61	1	Lobular	2.3	2	Positive	0.89	-1.88	-1.61	-0.94
16	73	22	Ductal	1.9	2	Negative	0.98	-1.28	-0.34	0.26
17	58	3	Ductal	9.0	2	Positive	0.96	-0.48	0.26	-0.42
19	68	13	Ductal	1.2	1	Negative	0.97	-2.18	-2.62	0.31
18^a^	67	11	Ductal	1.5	2	Micrometastasis	NA	NA	NA	NA
20	44	13	Ductulo-lobular	3.2	3	Positive	0.98	-0.12	0.01	0.19
21	71	20	Ductulo-lobular	1.7	3	Negative	0.95	-3.15	-1.69	0.26
22	53	5	Ductal	1.3	2	Negative	0.92	-2.81	-5.48	-0.08

^a^RNA in the core biopsy (CB) of this patient was degraded and, therefore, omitted from the study. COX, cyclooxygenase; NA, not available; PAI-1, Plasminogen Activator Inhibitor-1; ST, surgical tumor tissue specimen.
